# Breaking Liebig’s Law: An Advanced Multipurpose Neuromorphic Engine

**DOI:** 10.3389/fnins.2018.00593

**Published:** 2018-08-29

**Authors:** Runchun Wang, André van Schaik

**Affiliations:** The MARCS Institute, Western Sydney University, Sydney, NSW, Australia

**Keywords:** neuromorphic engineering, spiking neural networks, neural engine, FPGA, spike timing dependant plasticity, spike timing dependant delay plasticity

## Abstract

We present a massively-parallel scalable multi-purpose neuromorphic engine. All existing neuromorphic hardware systems suffer from Liebig’s law (that the performance of the system is limited by the component in shortest supply) as they have fixed numbers of dedicated neurons and synapses for specific types of plasticity. For any application, it is always the availability of one of these components that limits the size of the model, leaving the others unused. To overcome this problem, our engine adopts a unique novel architecture: an array of identical components, each of which can be configured as a leaky-integrate-and-fire (LIF) neuron, a learning-synapse, or an axon with trainable delay. Spike timing dependent plasticity (STDP) and spike timing dependent delay plasticity (STDDP) are the two supported learning rules. All the parameters are stored in the SRAMs such that runtime reconfiguration is supported. As a proof of concept, we have implemented a prototype system with 16 neural engines, each of which consists of 32768 (32k) components, yielding half a million components, on an entry level FPGA (Altera Cyclone V). We verified the prototype system with measurement results. To demonstrate that our neuromorphic engine is a high performance and scalable digital design, we implemented it using TSMC 28nm HPC technology. Place and route results using Cadence Innovus with a clock frequency of 2.5 GHz show that this engine achieves an excellent area efficiency of 1.68 μm^2^ per component: 256k (2^18^) components in a silicon area of 650 μm × 680 μm (∼0.44 mm^2^, the utilization of the silicon area is 98.7%). The power consumption of this engine is 37 mW, yielding a power efficiency of 0.92 pJ per synaptic operation (SOP).

## Introduction

Neurobiological processing systems can easily outperform the most up-to-date computers at robustly accomplishing real-world tasks such as sensory-motor tasks. It still remains largely unknown how biological brains can achieve this with slow, stochastic, and heterogeneous computing elements (Wang, 2013). In the late 1980’s, Caver Mead introduced neuromorphic engineering – a multidisciplinary approach to develop a new generation of computing technologies, building sensory and processing systems using very large scale integration (VLSI) circuits inspired by principles of the biological nervous system.

Since the first silicon neuron proposed by Mahowald and Douglas (1991), significant progress has been made and various designs based on analog, digital and mixed-signal VLSI have been developed (Wang, 2013). Examples include the Neurogrid project which emulates one million neurons connected by six billion synapses (Boahen, 2006), the BrainScaleS project, a wafer-scale neural network, which contains 384 analog network chips for a total of 40M synapses and 200K neurons (Schemmel et al., 2008, 2010), the SpiNNaker project (Furber et al., 2014), which uses ARM processors to run software neural models and a 48-node SpiNNaker board is capable of simulating 250,000 neurons and 80 million synapses in real time, the IBM TrueNorth chip (Merolla et al., 2014) that is capable of running one million leaky-integrate-and-fire (LIF) neurons in real time. The HiAER-IFAT system has five FPGAs and four custom analog neuromorphic integrated circuits, yielding 262k neurons and 262M synapses. The full-size HiAER-IFAT network has four boards, each of which has one IFAT module, serving 1M neurons and 1G synapses.

It is generally accepted that learning in the brain arises from synaptic modifications. Hence, plastic synapses, which can adapt their gain according to one or more adaptation rules, play a vital role in neural systems. The STDP algorithm (Gerstner et al., 1996; Magee, 1997; Markram et al., 1997; Bi and Poo, 1998), is one of the adaptation rules observed in biology. It modulates the weight of a synapse based on the relative timing between the pre-synaptic spike and the post-synaptic spike. Due to its importance, many hardware implementations of this STDP learning rule have been proposed (Chicca et al., 2003; Bofill-i-petit and Murray, 2004; Indiveri et al., 2006; Häfliger, 2007; Koickal et al., 2007; Mitra et al., 2009; Giulioni et al., 2012).

Besides weight adaptation, some observations suggest that the propagation delays of neural spikes, as they are transmitted from one neuron to another, may be adaptive (Stanford, 1987). Axonal delays seem to play an important role in the formation of neuronal groups and memory (Izhikevich, 2006). In our previous work (Wang et al., 2011b, 2012), a delay adaptation algorithm, STDDP, inspired by STDP was developed to fine-tune delays that had been programmed into the network. We have successfully implemented the STDDP learning rule on hardware (Wang et al., 2011a,b, 2012, 2013a).

However, all these neuromorphic hardware systems are subject to Liebig’s law, because they have a fixed number of neurons and a fixed number of synapses of each specific type of plasticity that was implemented. In most cases, these dedicated components are all hardwired, e.g., one neuron’s learning synapses can’t be used by other neurons. In almost all cases, for any implemented architecture, there is one type of component that will be used up first, leaving the others unused. To address this, we have designed a system where each component can be reconfigured to perform different roles. We have previously presented a hardware implementation of a synaptic plasticity adaptor that is capable of performing either STDP or STDDP (Wang R. et al., 2014; Wang et al., 2015). However, that system does not support online reconfiguration: recompiling of the design is required to switch functions. More importantly, this adaptor can only perform learning rules and it can’t be used to implement neurons. Here, we report its follow up work: a massively-parallel scalable multi-purpose neuromorphic engine.

## Materials and Methods

### Strategies

Traditionally, the neuromorphic field views its computing devices as hardware designs that are inspired by the biological brain. The latter differs drastically from general purpose, von Neumann computing architectures. The fundamental features of biological neural networks, such as (1) massively parallel and distributed components, (2) integrated memory and compute, (3) spiking, (4) local learning rules, and (5) sparse and asynchronous communications between components, etc., are usually accepted as a whole-sale package, taken altogether or none. But these properties are actually very distinct. Partial adoption of them is thus likely to yield optimal solutions for solving certain problems.

**FIGURE 1:**
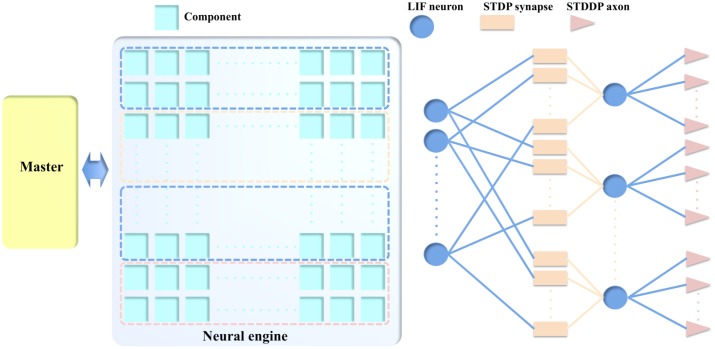
The example usage of the components. In this example, one and only one type of components are configured as LIF neurons, STDP synapses, and STDDP axons. They are used to build the different part of the neural network.

We chose to abstract away peculiarities of neuronal and synaptic mechanics and regard components as generally configurable state machines that communicate in an event-based manner, while maintaining a non-von Neumann distributed design. Based on this strategy, we propose a single component capable of performing different functions (see **Figure 1**) as a compelling case for an educated partial selection of features to put in hardware. This concept uses the fact that neurons, synapses, and axons all require local memory, such as SRAM, around which a multiple-purpose component can be built. The SRAM is the most important resource that will be shared by the LIF neurons, STDP adaptors and STDDP adaptors. **Figure 1** exploits the benefits proposed in this paper related to reconfiguration:

When one component is configured as a neuron, it receives input from other neurons via its synapses onto its cell body (the soma). The soma performs a leaky integration of the post-synaptic currents (PSCs, generated by its synapses) to calculate its membrane potential and generates an output spike when the membrane potential passes a threshold, after which the membrane potential is reset and enters a refractory period.

When one component is configured as an STDP adaptor, it performs the STDP learning rule by receiving pre- and post-synaptic spikes from a pre- and post-synaptic neuron, respectively. Its output, a weighted pre-synaptic spike is sent to the synapse of the post-synaptic neuron for generating a PSC. When one component is configured as an STDDP adaptor, it receives the same inputs, but its output is a pre-synaptic spike that has been delayed according to the stored delay value for this neuron-to-neuron connection. Note, multiple different types of adaptors can be combined and connected to a single neuron.

The communications between the components are controlled by a Master module, which, on receiving a spike, will remap the address of the pre-synaptic neuron to the address of the post-synaptic neuron. This strategy provides great flexibility, as a system can be configured to perform multiple tasks without needing to change its own hardware, simply by changing the parameters and the connections between the components in memory. More importantly, this strategy allows the neural network to be highly optimized such that all the components can be effectively used.

Implementing a synapse array that is separate from the neurons is not a new idea. This structure was first proposed in the IFAT project (Vogelstein et al., 2007). It provides great flexibility and reconfigurability, as a hardware platform can be configured for simulating different types of neural networks without needing to change its own structure, simply by connecting the synapses to the appropriate neurons. Due to these advantages, in spite of the overhead that stems from the communication from the plastic synapses to each of the neurons, this design strategy has been widely used in state-of-the-art mixed-signal neuromorphic systems (Qiao et al., 2015; Moradi et al., 2018) and the recent fully digital ones (Merolla et al., 2014; Davies et al., 2018; Frenkel et al., 2018). This is made possible because these systems all employ high-speed digital circuits that can easily handle this overhead.

### Modeling

#### Conductance-Based Neuron

Rather than using a mathematical computational model, the physical neuron is efficiently implemented using a stochastic conductance-based model. It has a soma and a single PSC generator that can generate both excitatory PSCs (EPSCs) and inhibitory PSCs (IPSCs). Its function is expressed by the following equation:

(1) $$\mathrm{PSC}(t+1)=\mathrm{PSC}(t)\times \frac{\tau_{\mathrm{psc}}}{\tau_{\mathrm{psc}}}+r(t)$$

where *t*+1 represents the index of the current time step, *PSC*(*t*) represent the value of the PSC (from memory), which is a 4-bit number normalized in the range [−1,1]. τ*_PSC_* represents the time constant, e.g., 10 ms, controlling the speed with which the *PSC* will decay to 0 exponentially. The PSC generator will use τ_EPSC_ and τ_IPSC_ for EPSC and IPSC respectively. *r*[t] is a random number drawn from a uniform distribution in the range (0,1) and is different at different time steps. Note, the previous equation is only for implementing the exponential decay of the PSC. On arrival of an input spike, the PSC will be updated using the following equation:

(2) PSC=PSC+W

where *W* represents the synaptic weight of the pre-synaptic spike. *W* is also a 4-bit number normalized in the range [−1,1].

The soma is also a stochastic conductance-based model similar to the PSC generator. Its function is expressed similarly by the following equation:

(3) $$\begin{array}{l} V_{\text{mem}}(t+1)=V_{\text{mem}}(t)\times \frac{\tau_{\text{soma}}}{\tau_{\text{soma}}+1}\\ \;+r(t)+g_{\text{psc}}\times \mathrm{PSC}(t+1) \end{array}$$

where *V*_mem_(*t*) represents the previous value of the membrane voltage (from the memory). The soma has two states: an active state and a refractory state. The soma only integrates *PSC(t+1)* when it is in its active state. When its membrane voltage is below a configurable resting value, the soma is in its refractory state and the PSC will be discarded. τ_soma_ represents the time constant controlling the speed with which *V*_mem_ will decay to the resting value exponentially. The soma will use τ_mem_ and τ_rfc_ for τ_soma_ in its active and refractory period, respectively. *r*[t] is another random number drawn from a uniform distribution in the range (0,1). *g*_psc_ is a configurable parameter and will be addressed in detail in Section “Time-Driven Module”. The soma will generate a post-synaptic spike when *V*_mem_ overflows. *V*_mem_ will be reset, when there is an overflow/underflow caused by EPSC or IPSC, respectively.

#### STDP Learning Rule

**FIGURE 2:**
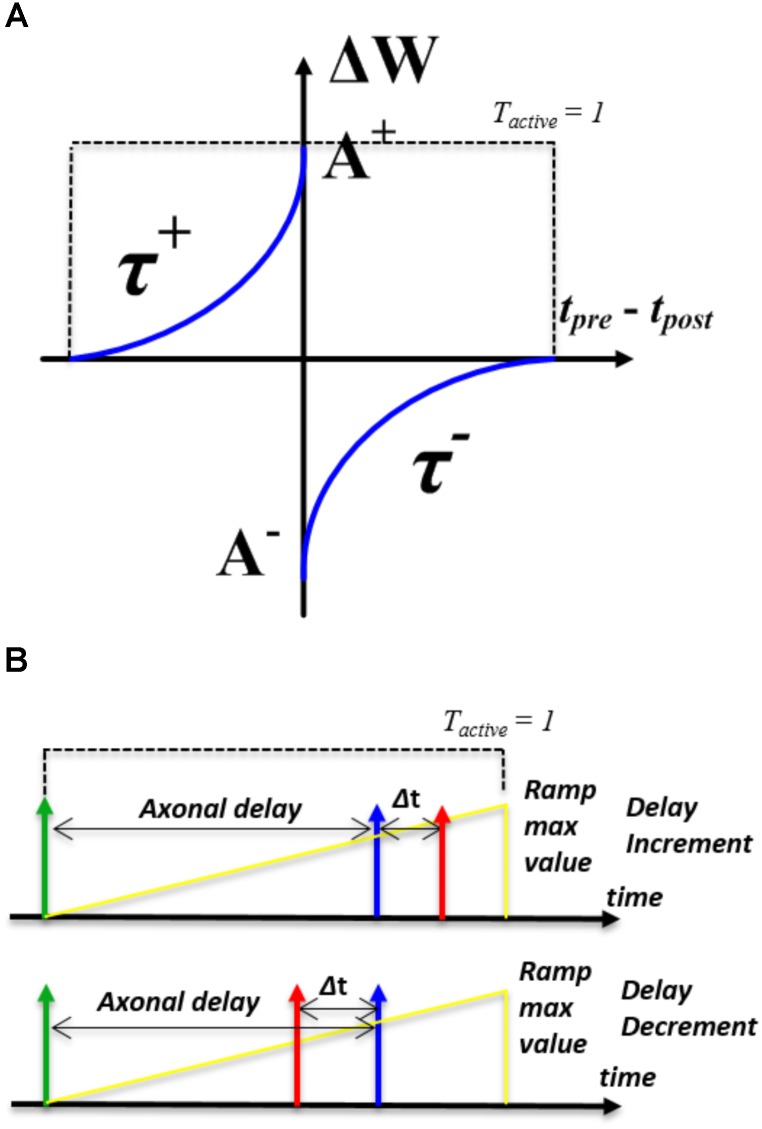
Illustration of learning rules. **(A)** the STDP modification function; **(B)** the STDDP learning rules.

In the STDP algorithm (see **Figure 2A**), the amount and direction of modification of the weight are determined by the time between the arrival of the pre- and post-synaptic spike. To obtain this time difference, we need to know when the pre- and post-synaptic spike arrive. This is implemented by introducing a time window generator, which will be set to its maximum value by either spike and then decay to zero exponentially. The time window is “active” until it reaches zero and the time at which the second spike arrives is encoded by the value of the time window generator. The direction of the update will depend on whether the second spike is from a pre-synaptic or a post-synaptic neuron. We assume that no adaption will be carried out if the pre- and post- synaptic spikes arrive simultaneously. Note, the time window needs to decay to zero before it can be started again. Therefore, multiple weight updates will happen if multiple spikes arrive within an active time window.

We have implemented two simplified modification rules. In the first one, the amount of synaptic modification is summarized by the following standard exponential STDP equations:

(4) $$\Delta w=\{\begin{array}{l} A^{+}\exp(\Delta t/\tau_{+}),\;\mathrm{if}\;T_{\text{active}}=1\;\mathrm{and}\;\Delta t<0\\ -A^{-}\exp(\Delta t/\tau_{-}),\;\mathrm{if}\;T_{\text{active}}=1\;\mathrm{and}\;\Delta t>0 \end{array}$$

where, Δ*w* is the modification of the synaptic weight, Δ*t* is the arrival time difference between the pre- and post-synaptic spike. *T*_active_ is a Boolean value that indicates the time window generator is active (see the dashed line in **Figure 2A**). *A*^+^ and *A^−^* determine the maximum amounts of synaptic modification for each spike pair. τ_+_ and τ*_-_* are the time constants and control the rate of decay for the potentiation and depression portions of the curve, respectively. The parameters τ_+_, τ*_-_*, *A^+^* and *A^−^* are all configurable.

A second version changes the weight by a fixed value as long as the second spike arrives during the active time window and is summarized by the following equations:

(5) $$\Delta w=\{\begin{array}{l} +\mathrm{step},\;\mathrm{if}\;T_{\text{active}}=1\;\mathrm{and}\;\Delta t<0\\ -\mathrm{step},\;\mathrm{if}\;T_{\text{active}}=1\;\mathrm{and}\;\Delta t>0 \end{array}$$

where *step* is also a configurable value. This modification rule has been proven to be functional in our previous work (Wang et al., 2015).

#### STDDP Learning Rule

The STDDP learning rule is shown in **Figure 2B**. After a pre-synaptic neuron fires (the green spike in **Figure 2B**) there is an axonal delay before the delayed pre-synaptic spike (the blue spike in **Figure 2B**) is sent to the post-synaptic neuron. If the post-synaptic spike (the red spike in **Figure 2B**) is not simultaneous with the delayed pre-synaptic spike, we adapt the axonal delay by increasing or decreasing it by a small amount. This procedure is repeated until the delayed pre-synaptic spike occurs simultaneously with the post-synaptic spike.

To obtain the time difference between the pre- and post-synaptic spikes, we will use a ramp generator to generate the axonal delay. The ramp generator will be started by a pre-synaptic spike (the green spike in **Figure 2B**). Once started, the ramp (the yellow line in **Figure 2B**) will be increased on each time step and it is “active” until it reaches the maximum value. Further pre-synaptic spikes arriving during the active period will be discarded. The modification of the axonal delay will only be performed following the arrival of a post-synaptic spike: when the post-synaptic spike arrives, if the ramp is active and its value is greater/less than the axonal delay, then there is a decrease/increase of the axonal delay. While if the ramp is not “active,” there will be no modification.

We have implemented two modification rules. The first one is called linear proportional modification rule, the modification of the axonal delay Δ*d* is summarized by the following equations:

(6) $$\Delta d=\{\begin{array}{l} A^{+}\Delta t,\;\mathrm{if}\;T_{\text{active}}=1\;\mathrm{and}\;\Delta t<0\\ -A^{-}\Delta t,\;\mathrm{if}\;T_{\text{active}}=1\;\mathrm{and}\;\Delta t>0 \end{array}$$

where Δ*t* is the arrival time difference between the pre- and post-synaptic spike. *T*_active_ is a Boolean value that indicates the time window generator is active (see the dashed line in **Figure 2B**). *A*^+^ and *A^−^* determine the maximum amounts of axonal delay modification for each spike pair. No delay modification will be performed if the delayed pre-synaptic spike and post-synaptic arrive simultaneously. The second rule is to change the value of the axonal delay by a fixed value and is summarized by the following equations:

(7) $$\Delta d=\{\begin{array}{l} +\mathrm{step},\;\mathrm{if}\;T_{\text{active}}=1\;\mathrm{and}\;\Delta t<0\\ -\mathrm{step},\;\mathrm{if}\;T_{\text{active}}=1\;\mathrm{and}\;\Delta t>0 \end{array}$$

where *step* is also a configurable value. We have tested these modification rules in previous work (Wang et al., 2013b).

### Hardware Implementation

#### Topology

To perform complicated real-world problems, e.g., pattern recognition on images, spiking neural networks will require a very large number of neurons and synapses, particularly plastic synapses. Hence, the footprint of our generic component needs to be as small as possible. To achieve this requirement, the design choice, e.g., analog/digital and synchronous/asynchronous, varies for different CMOS technology nodes.

With state-of-the-art CMOS technologies, we can easily time-multiplex a single physical component to simulate many virtual (or time-multiplexed, TM) components, with each one updated every millisecond. Since powers of two are preferable to optimize memory use for storage of the neural network variables, the neural engine is configured to time-multiplex 2,048 (2k) virtual components. The sizes of neural states of all the three components are the same: eight bits (**Table 1** shows the arrangement). They are stored in SRAM, for which we use dual-port devices, since the TM approach needs to write and read them simultaneously. The footprint of the physical component is negligible compared to the area of the SRAMs, which are far more area efficient than logic gates in state-of-the-art CMOS technologies (Wu et al., 2009), e.g., 28 nm and the ones afterwards. We will demonstrate this in more details in Section “Hardware Evaluation.”

**Table 1:** Arrangement of the neural states.

	Bit[7:4]	Bit[3:0]
LIF neuron	Membrane voltage (*V_mem_*)	PSC
STDP adaptor	The exponential time window. Bit[7] indicates the polarity of the time window (0 and 1: triggered by pre- and post-synaptic spike). Bit[6:4] are the value of the exponential time window.	Bit[3]: single active signal of the pre-synaptic spike Bit[2:0]: synaptic weight (positive only)
STDDP adaptor	Ramp value	Axonal delay

**FIGURE 3:**
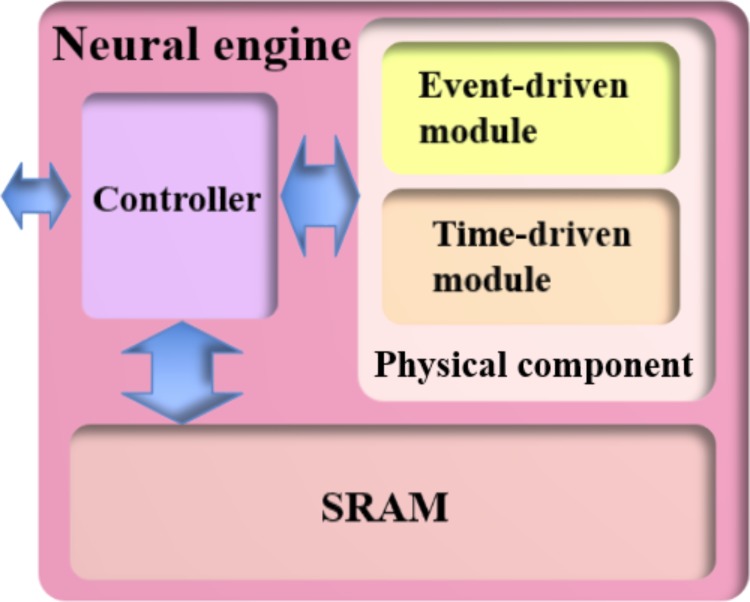
A naïve implementation of the neural engine. The physical component computes the data for one virtual component module in one clock cycle. The controller provides the physical component with its input data, which is read from the SRAM, and writes the computed output data to the SRAM.

**Figure 3** shows a naïve implementation of the neural engine. It consists of a controller, an SRAM, and a physical component, which implements the LIF neuron, the STDP adaptor, and the STDDP adaptor. They are all implemented on an FPGA, which runs at 200 MHz. The controller is the central part of the engine as it informs the other parts as to which TM component is currently being processed. The controller receives pre- and post-synaptic spikes from the Master and assigns them to the corresponding (TM) components according to their addresses.

#### Advanced Time-Multiplexing Approach

Time multiplexing introduces the requirement of decoupling writing new events from reading out current events, since incoming events for a TM neuron will most likely not arrive at the exact time step this neuron is being processed. In previous implementations (e.g., Cassidy et al., 2011), a buffer is employed to queue incoming events which are then sent to the TM neurons at the appropriate time step. This type of solution is not very friendly for hardware implementation since it uses significant memory: all the events that arrived within the last time step, e.g., the last 1 ms, have to be stored. When this buffer is full, the system will have to stop receiving new spikes or it will lose spikes.

To overcome this bottleneck, we adopted an advanced time-multiplexing approach, which splits the time slots into two groups: time-driven slots and event-driven slots, because the tasks that need to be performed by the components can be classified into event-driven tasks and time-driven tasks (see **Table 2**).

**Table 2:** Functions of the physical component.

	Time-driven tasks	Event-driven tasks
LIF Neuron	(1)Apply the leak to the PSC and the membrane voltage. (2)Integrate the PSC onto the membrane voltage. (3)Generate a post-synaptic spike when the membrane voltage reaches its maximum value. (4)Reset the membrane voltage after the spike generation.	(1) Update the PSC with the incoming pre-synaptic spikes.
STDP adaptor	(1)Apply the leak to the time window for the exponential decay. 2. Generate a weighted pre-synaptic spike with the synaptic weight when there is a pre-synaptic spike (the single indication bit is active).	(1) Start the time window when there is an incoming spike. (2) If it is a pre-synaptic spike, set a single active bit for indication. (3) Update the synaptic weight according to the STDP rule when an alternative spike arrives within an active time window.
STDDP adaptor	(1) Increase the ramp value by 1 when the ramp is active. (2) Generate a delayed pre-synaptic spike when the ramp value exceeds the axonal delay.	(1) Start the ramp when there is a pre-synaptic spike. (2) Update the delay value according to the STDDP rule when a post-synaptic spike arrives within an active ramp.

**FIGURE 4:**
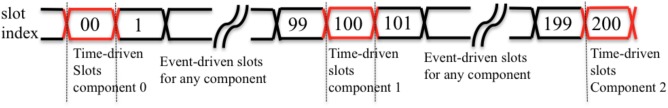
Timing diagram of the advanced time-multiplexing approach.

The controller will send incoming events to the destination components, e.g., neurons and adaptors, in the event-driven slots such that the requirement for holding huge amounts of incoming events is eliminated. In the time-driven slot (for each component), we will perform the time-driven tasks. **Figure 4** shows a simplified timing diagram of this advanced time-multiplexing approach. For simplicity, let’s assume that each component takes only one clock cycle to process one event-driven task or one time-driven task. In this example, the time-driven slot takes place every 100 clock cycles. Between any two of time-driven slots, there are then 99 event-driven slots that can be used by any component for event-driven tasks. As this neural engine is configured to have 2k components, for each component, the time between two time-driven tasks will be (2k × 100)/200 MHz ≈ 1 ms, which is needed for real time simulation. On the other hand, the incoming spikes will be processed immediately in the event-driven slots.

For applications of neural networks that need to process higher firing rates, this method would still work by trading off event-driven slots for multiplexing ratio and network size. For instance, we can increase the number of event-driven slots (between two time-driven slots) from 99 to 199 by reducing the size of the neural engine from 2k components to 1k components. We will address our design choice of 2k components in Section “Prototype System.”

Incoming events cannot arrive at the neural engine during the time-driven slots. This constraint can be easily guaranteed by the Master, which will hold the spikes for that time-driven slot, since the neural engine is deliberately designed to be a slave device and both Master and the neural engine are synchronous digital designs.

Since the need of a large buffer has been eliminated, this method significantly reduces memory usage, enabling more components to be implemented. Moreover, it reduces the latencies in communication that would result from using a buffer. In a buffered implementation, incoming spikes wait in the buffer until their destination TM neuron’s turn arrives. Such a latency can be of the order of hundreds of microseconds. The communication overhead between the Master and the neural engine will barely affect the performance of the system.

#### Physical Component

To comply with the advanced time-multiplexing approach, the physical component is divided into two sub-modules: the event-driven module and the time-driven module. These two modules are both physically implemented and do not communicate with each other directly. The event-driven module receives the incoming events (from the controller) and uses them to update the neural states. The time-driven module generates events (post-synaptic spikes, delayed pre-synaptic spikes and weighted pre-synaptic spikes) from the neural states. The controller of the engine will schedule the progress of the time-multiplexing approach. For both modules, the DSPs are deliberately designed to be shared by the LIF neuron and the STDP adaptor.

##### Time-driven module

**FIGURE 5:**
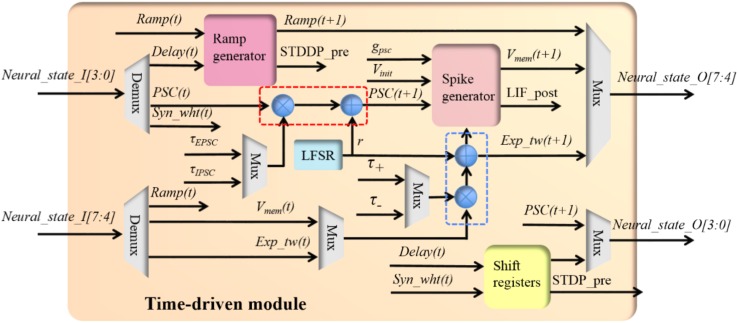
The structure of the time-driven module.

The time-driven module performs the time-driven functions of all three components (see **Table 1**). Since at any given time, it only needs to execute one and only one component’s functions, it is not necessary to implement all the functions separately. Instead, they are efficiently implemented in a single module. **Figure 5** shows the structure of the time-driven module, the core of which are two multipliers for implementing exponential decays. The input neural states will be processed by any of the three components, i.e., independently of the configuration of the time-driven module. Multiplexers are employed to select the correct outputs from the time-driven module to the controller block.

The time-driven module has a 5-stage pipeline without halt: the first cycle selects the parameters and input neural states; the second and third cycle perform the multiplication and addition, respectively; the fourth cycle performs the spike generation and the last cycle outputs neural states. Each TM component will access each computing unit such as the adder and the multipliers for only one clock cycle. On one clock cycle, the adder and the multipliers are all being used, but by different TM components. Therefore, the overhead of the pipeline is negligible: 10 clock cycles, the first 5 cycles for setting up the pipeline and the last 5 cycles for waiting for the last TM component to finish processing.

When configured to be working in the LIF neuron mode, the module performs the time-driven functions for the PSC generator and the soma. The time-driven function [equation (1)] of the PSC generator is implemented with a multiplier and an adder (the red dashed rectangle in **Figure 5**). The random number *r* in equation (1) is generated by a linear feedback shift register (LFSR). The output of the PSC generated will be sent out (to the controller) and to the soma.

The time-driven function of the soma is implemented with a multiplier, an adder (the ones in the blue rectangle) and a spike generator. A naïve implementation of the function *g*_psc_ × *PSC(t+1)* in equation (3) is to use a multiplier. Since in a digital design significant implementation advantages can be gained if powers of two arithmetic can be used for multiplication and division, we modified *g*_psc_ to be a fixed parameter (3 bits for eight values: 1/32, 1/16, 1/16, 1/8, 1/2, 1, 2, and 4). This function is efficiently implemented in the spike generator using a selector and a shift circuit. The spike generator performs the function of generating the post-synaptic spike (LIF_post, 1 bit) and resetting the membrane voltage to zero. The outputs of the spike generator will be sent to the controller.

When configured to be working in the STDP adaptor mode, the module implements the exponential decay of the time window and generates the weighted pre-synaptic spike (STDP_pre, 4 bits). For the sake of saving hardware resources, we deliberately designed the circuit such that the implementation of the exponential decay shares the same multiplier and adder (in the blue rectangle) used by the soma. The time constants will be selected based on the working mode of the module. Its product [exp_tw(*t*+1)] will be sent to the controller as the four MSBs bits of the output neural state via the multiplexer. The STDP_pre is generated by moving the lowest significant four bits of the input neural state to the output. This is done using shift registers to comply with the pipeline delays. The four LSBs of the output neural state are processed in the same way as the STDP_pre with its single active bit (bit[3], in **Table 2**) being cleared.

When configured to be working in the STDDP adaptor mode, this module implements the ramp generator and increases the ramp value (the four MSBs of the input neural state) by one each time step, until the ramp value reaches the maximum value (0×F). The module will generate the delayed pre-synaptic spike (STDDP_pre, 1 bit) when the ramp value exceeds the axonal delay (the four LSBs of the input neural state), which will be sent out via the shift registers to comply with the pipeline.

##### Event-driven module

**FIGURE 6:**
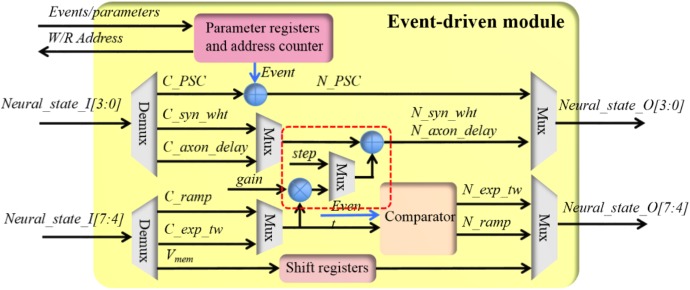
The structure of the event-driven module.

The event-driven module has also been implemented to perform all event-driven tasks (see **Table 1**) of all three components in a single block. **Figure 6** shows the structure of the event-driven module, which has a 6-stage pipeline without halt: the first cycle selects the parameters and input neural states; the second and third cycle perform the multiplication and addition, respectively; the fourth and fifth cycle performs the modification of the neural states and the last cycle outputs neural states. For each incoming event, its address will be used by an address generator for fetching the neural state from the SRAM (via the controller). Similarly to the time-driven module, the input neural state will be processed by all three components, irrespective of the module’s configuration. As before, multiplexers are employed to select the correct outputs to the controller.

As a pipelined design, each incoming event will access each computing unit such as the adder and the multipliers for only one clock cycle. On one clock cycle, the adder and the multipliers are all being used but by different events. In other words, this event-driven module is virtually processing one event per clock cycle. For instance, if the controller moves in 1,000 consequent events, the overall processing time is 1000 + 6 + 6 = 1012 clock cycles: the first six cycles for setting up the pipeline and the last six cycles for waiting the last event to be processed. To comply with this pipeline, the controller will write the parameters, e.g., the amount of the upcoming events and the working mode, into the parameter registers first. After that, the controller will move in the required amount of events while pipeline pause is allowed during this movement. The address counter will generate the read and write address for accessing the SRAM according the parameters.

When configured to be working in the LIF neuron mode, the incoming pre-synaptic events will be used to update the PSC. The current PSC (C_PSC, from the SRAM) will be incremented by the synaptic weight of this event. Both the PSC and the synaptic weight are signed numbers, to avoid subtraction operations. The output (N_PSC) will be sent to the controller, and then written into the SRAM. If this event is from an STDP adaptor, the event contains the synaptic weight (see **Table 2**). Otherwise this synaptic weight is a configurable parameter: a fixed or random weight. This parameter needs to be configured by the controller before moving the events. The membrane voltage will be simply sent out via shift registers, again for complying with the pipeline.

When configured to be working in the STDP adaptor mode, the incoming event and the current value of the time window (C_exp_tw) will be sent to a comparator. If this time window is not active, its next value (N_exp_tw) will be set to the maximum value (0 × 7). For the time window started by pre- and post-synaptic spikes, the polarity value is 0 × 0 and 0 × 1, respectively. Otherwise, the value of this time window will not change (N_exp_tw = C_exp_tw).

In the meanwhile, the synaptic weight will be updated according to the rules in equations 4 and 5 presented in Section “STDP Learning Rule.” They are efficiently implemented with a multiplier and two adders (in the red rectangle in **Figure 6**). Subtraction operations are avoided by using signed numbers such that no comparing circuit is needed. A multiplexer is introduced for choosing different update rules. The *gain* and the *step* correspond to *A* and *step* in equations 4 and 5, respectively. These parameters need to be configured by the controller before moving the events.

When configured to be working in the STDDP adaptor mode, the incoming event and the current value of the ramp (C_ramp) will be sent to the comparator. If the ramp is not active (C_ramp = 0 × F) and the incoming event is a pre-synaptic event, its next value (N_ramp) will be set to the initial value (0 × 0). Otherwise, the value of the ramp will not change (N_ramp = C_ramp).

In the meanwhile, the axonal delay will be updated according to the equations 6 and 7 presented in Section “STDP Learning Rule,” if the incoming event is a post-synaptic event. The same multiplier and adders in the red rectangle (**Figure 6**) are used for this. Multiplexers are introduced to choose different working modes. The *gain* and the *step* correspond to *A* and *step* in equations 6 and 7, respectively. Again, these parameters need to be configured by the controller before moving the events.

Besides these functions, this event-driven module is capable of programming the value of the synaptic weight and the axonal delay with user defined values. This is essential for algorithm development.

#### Multi-Component Engine

Two methods are used to scale up the neural engine with significant reduction of hardware cost. A naïve implementation of the neural engine (see **Figure 3**) has one controller and one physical component. The first method is to implement one neural engine with one controller and multiple physical components that run in parallel, since these physical components operate in the same way with the same interface. This reduces not only the hardware costs but also the amount of the events that need to be moved by the Master, as components nearby are very likely to receive the same events. Otherwise, we will have to replicate the same events for each component.

The second method achieves a significant saving of hardware resources by sharing one time-driven module across these parallel physical components, since the number of event-driven slots is much higher than the number of time-driven slots. In our system, the ratio between the event-driven slots and the time-driven slots is 99:1, hence theoretically one time-driven module can be used by 99 physical components, each of which has its own event-driven module. However, it is quite difficult to achieve this ratio in a practical design, since it will introduce significant routing issues: the controller has to communicate with all of these 99 physical components. These routing issues will also make it almost impossible to meet the critical timing requirements. As a trade-off, the neural engine is designed to have 16 physical components (yielding 16 × 2k = 32k components) that share one time-driven module.

**FIGURE 7:**
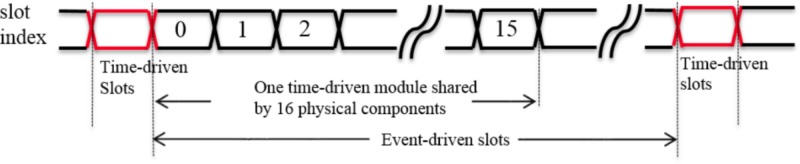
Timing diagram of the sharing time-driven module.

In this method (see **Figure 7**), the neural states (16 × 8 = 128 bits) for the time-driven module are latched in the first time-driven slot. Then this time-driven module will process these neural states in the following 16 clock cycles (ignoring the overhead of the pipeline). The updated neural states and events that have been generated will be latched and written into the SRAM by the controller during the next time-driven slot. Since the time-driven module and event-driven module are deliberately designed to be independent of each other, the event-driven modules can continue to receive and process events during these 16 clock cycles.

#### Controller

To ensure the Master has full control of the processing progress, the controller operates in an instruction-driven manner. It receives the start instruction from the Master and then runs for a number of time slots, which need to be configured by the Master in advance. After this run, it will wait for the next start instruction. Besides the number of the time slots, the Master also needs to send the parameters for the time-driven module before one run starts. This scheme not only ensures the Master’s full control of the processing progress, but also provides great flexibility as the neural engine can be easily reconfigured with different parameters for different applications.

**FIGURE 8:**
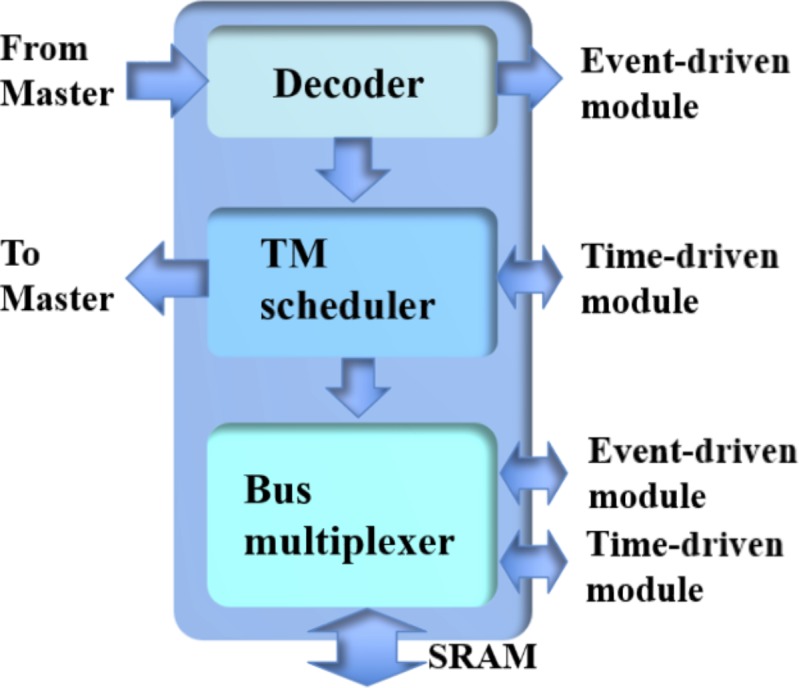
The structure of the controller.

**Figure 8** shows the structure of the controller, which consists of a decoder, a TM scheduler, and a bus multiplexer. The decoder is for decoding the instructions from the master and it will pass events from the Master to the event-driven modules. Its outputs are the parameters required for scheduling the simulation by the TM scheduler, which are implemented with two counters: one for the event-driven slots and one for the time-driven slots.

The TM scheduler will control the bus multiplexer, which multiplexes the neural states from/to the time-driven and event-driven modules. Since the SRAM is a dual-port device, the multiplexing operation can be easily handled. The events generated will be sent to the Master by the TM scheduler, since the address of the synchronous AER protocol is indeed the value of the counter for the time-driven slots.

## Results

### Prototype System

**FIGURE 9:**
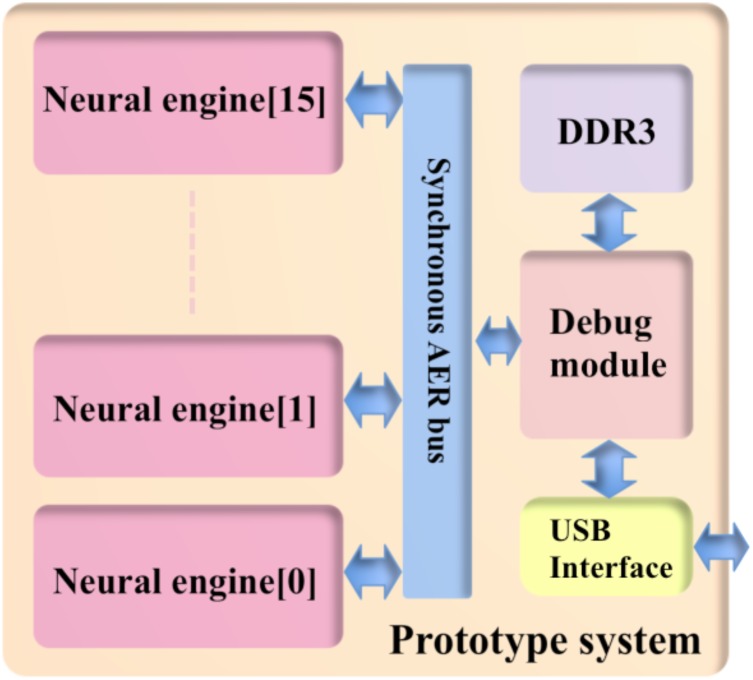
Structure of the prototype system. To achieve the maximum utilization of the hardware resources, 16 neural engines are placed parallel in the system.

As a proof of concept, we implemented a prototype system on a Cyclone V FPGA, which is hosted on a Terasic SoC kit. We can incorporate more than one neural engine into the system in order to increase capacity. Each neural engine is connected in parallel in the system (see **Figure 9**). The number of TM components implemented by one physical component will increase linearly with the amount of available on-chip SRAM, as long as the multiplexing rate keeps the time resolution acceptable, i.e., generally better than 1 ms, while more engines will use more logic gates (ALMs). The timing requirement will become quite critical when the utilization becomes high, e.g., 90% of the logic gates and SRAMs on an FPGA, due to the difficulties in routing.

We chose to use 2k TM components per engine, since it is a good balance between the number of the physical components and the multiplexing rate. It achieves a good time resolution and a high utilization of the available hardware recourses on FPGA. We integrated 16 neural engines in the system, yielding 16 × 32k = 512k (TM) components in total. The prototype system is developed to check correct operation of the functions of the neural engine, we used a debug module that is capable of generating stimuli and collecting test results, which will be stored in the DDR3 for buffering purposes. The prototype uses a high-speed USB interface to communicate with the host PC. **Table 3** shows the utilization of hardware resources on the FPGA. Note that this utilization of the prototype system includes the Altera IPs such as the ones for accessing external memories.

**Table 3:** Device utilization Altera cyclone 5CSXFC6D6F31C6.

	Adaptive Logic Modules (ALMs)	RAMs	DSPs
Neural engine	1250	256k bit	18
Event-driven module	946.4	N/A	16
Time-driven module	76.5	N/A	2
Controller	227.1	N/A	N/A
Prototype system	29212/41910	4.6 M bit/5.6 M bit	112/112^∗^

*^∗^One DSP slice can be used to implement three 9-bit fixed-point multipliers.*

The overall power consumption of this prototype system will not be determined by the type of the applications, or whether the components are configured as LIF neurons, learning synapses, or axons. Instead, power consumption mainly depends on the activity rate of the SRAM. The first reason for this is that the time-multiplexing approach will access the SRAM, even when there are no incoming events. Secondly, while the activity of the event-driven module is affected by the rates of the incoming events, this is not dependent on whether these events are for LIF neuron or adaptors. The higher the rate of the incoming events is, the higher the activity rate of the SRAM will be. We used PowerPlay, the power analysis tool provided by Altera to estimate the power dissipation of the FPGA, since a direct measurement of the FPGA’s power consumption is not possible on this development kit. PowerPlay estimated the power dissipation (see **Table 4**) from the netlist of the design, using the tool’s default settings and a 50% activity rate of the SRAM. The total power dissipation of the whole system is ∼1.95 W. The power dissipation per engine is 37.75 mW. Since there are 512k (TM) components, the power dissipation per component is 1.95 W/512k ≈ 3.7 μW.

**Table 4:** FPGA’s power dissipation estimated by PowerPlay.

Total power dissipation	1.95 W
I/O power dissipation	45.6 mW
Core static power dissipation	434.5 mW
Core dynamic power dissipation	1.48 W
One Neural engine dynamic power dissipation	37.75 mW
DDR interface dynamic power dissipation	67.16 mW
USB Interface dynamic power dissipation	20.61 mW
Debug module dynamic power dissipation	133.1 mW
Routing dynamic power dissipation^∗^	695.3 mW

*^∗^This item includes all the dynamic power dissipation caused by the routing of the signals (wires) between modules.*

Since this engine runs at 200 MHz and time-multiplexing rate is 2k (with a time slot of 100 clock cycles), the engine is capable of performing 200 M × (99/100) × 16 ≈ 3.2 G synaptic operations (SOPs) per second. The prototype system with 16 engines is therefore capable of performing 16 × 3.2G = 51.2 G SOPs per second. This FPGA prototype system achieves a power efficiency of ∼38 pJ per SOP.

The results presented here will focus on the capability of the neural engine, since our goal was to develop a flexible hardware implementation capable of performing the functions of a LIF neuron, a learning synapse, and an axon. Therefore, we did not test algorithms specifically developed for real-world applications, such as for pattern recognition. Instead, we verified the capability of the neural engine configured as a LIF neuron, a learning synapse, and an axon with common benchmark problems. We used a time step of 1 ms throughout these tests.

### Performance of LIF Neuron

We verified the function of the LIF neuron by running an experiment in which all the components of the 16 neural engines were configured to work in the LIF neuron mode. For simplicity, the parameters of the neurons are all set to the same values (see **Table 5**). Owing to the stochastic approach, the neurons in one engine are heterogeneous, even when using identical parameters and are therefore capable of emulating the variance in biological neurons. There was no connection between these LIF neurons and a single external excitatory spike train with a Poisson rate of ∼20 Hz was connected to all the LIF neurons to mimic background activity. This setup ensured that all the neurons received the exact same input stimulus. The synaptic weight of this Poisson spike train was set to 0.375, which was barely enough to drive the neurons to fire (using the parameters in **Table 5**).

**Table 5:** Parameters of the LIF neuron.

τ_EPSC_	6 ms
τ_lPSC_	6 ms
τ_mem_	5.5 ms
τ_rfc_	3 ms
*g*_psc_	1

**FIGURE 10:**
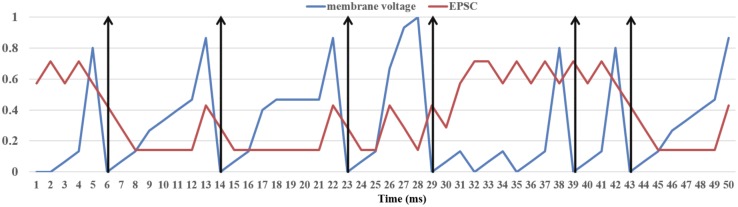
Dynamics of the LIF neuron. The membrane voltage and EPSC (of each 1 ms) were normalized in the range [0,1]. The vertical arrows are post-synaptic spikes, which will be generated when the membrane voltage exceeds 1.0.

**FIGURE 11:**
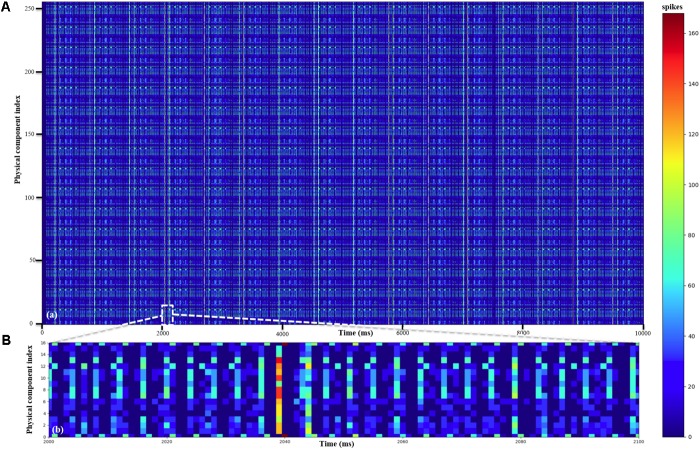
Performance of LIF neurons. **(A)** The mean firing rates of all the 256 physical components. **(B)** The mean firing rates of the 16 physical components of one neural engine (Neural_engine[0] in **Figure 9**) from 2 to 2.1 s.

We ran the experiment for 10 s. Since it is impossible to show a raster plot of half a million LIF neurons, we chose to show the simulation results in two parts. The first one (**Figure 10**) shows the dynamics of the membrane voltage and the EPSC of one randomly chosen neuron for 50 ms. It demonstrates that the physical component is capable performing the function of the LIF neurons. The second part (**Figure 11**) is the firing rates of the 256 physical components at each time step (1 ms). The firing rate was calculated by counting the spikes generated by all the 2k TM LIF neurons implemented with one physical component. The result (**Figure 11B**) also shows that the physical components are heterogeneous even when using the same parameters and are therefore capable of emulating the variance in biological neurons. The 16 neural engines in this prototype system were identical, therefore they produce identical results as **Figure 11** shows. When needed, we can program the seed for random number generation in each neural engine to be different, such that they would be heterogeneous too. We conducted this experiment for 10 runs with different seeds (for the generation of the external excitatory spike trains) and the mean firing rate of the 512k LIF neurons (of 10 runs) was ∼8.3 Hz

### Performance of STDP

We verified the function of the STDP adaptor by performing a balanced excitation experiment, based on the experiment run by (Song et al., 2000). Song et al. (2000) have shown that competitive Hebbian learning (Hebb, 1949) can be performed through STDP. The competition between the synapses induced by STDP will establish a bimodal distribution of the synaptic weights: either toward zero (*weak*) or the maximum (*strong*) values.

In this experiment, all the components of the 16 neural engines were configured to work in the STDP adaptor mode. Each adaptor was driven by an independent Poisson pre-and post-synaptic spike train. The firing rates of the pre-/post-synaptic spike trains of all the adaptors are the same. We have tested the system with two sets of firing rates. In the first one, the pre- and post-synaptic train was 10 and 10 Hz, respectively. In the second one, they were 10 Hz and 20 Hz, respectively. The adaptors start with a uniform positive weight distribution. We have conducted the experiment with both modification rules [equations (4) and (5)]. For both of them, we use τ_+_ = τ_-_ = 0.20 ms throughout. For the standard exponential modification rule, we use *A*^+^ = *A*^−^ = 1 throughout. For the fixed step modification rule, we use *step* = 1 throughout. For each configuration, we have conducted it for 10 runs with different seeds.

**FIGURE 12:**
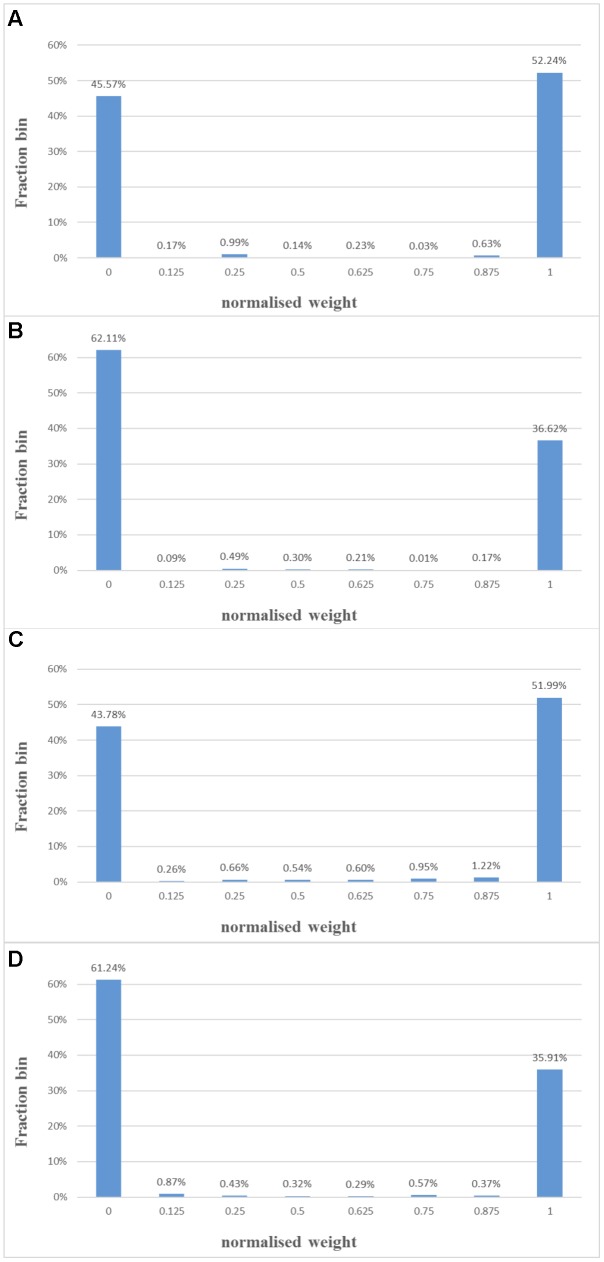
Balanced excitation experiment. **(A)** Mean weight distribution after 1 s of STDP with the standard exponential equations for a pre- and post-synaptic train with a rate of 10 and 10 Hz. The bimodal distribution of *strong* and *weak* weights is apparent; **(B)** same as **(A)**, but with a rate of 10 and 20 Hz. Now more weights are *weak* than *strong*; **(C,D)** same as **(A,B)**, but with a fixed adaptation step (set to 1 here).

After 1 s of simulation, the distribution of synaptic weights converges to a steady-state condition with bimodal distribution of *strong* and *weak* weights (see **Figure 12**, the mean weight distribution from 10 runs). Both modification rules confirmed the theoretical behavior: *for low input rates, more synaptic adaptors approach the upper limit, and for high input rates, more are pushed toward zero* (Song et al., 2000).

### Performance of STDDP

We verified the function of the STDDP adaptor by performing a polychronisation experiment, in which the axonal delays between the neurons are fine tuned such that these neurons can fire asynchronously but after traveling along axons, their spikes will arrive at a post-synaptic neuron simultaneously, causing it to fire in turn (Izhikevich, 2006). Polychronous Neural networks, which are based on this principle and are capable of storing and recalling quite complicated spatio-temporal patterns. In our previous work (Wang R.M. et al., 2014), we have concluded that providing a strong time-locked relationship is the most important requirement of a hardware implementation of a polychronous network. This is indeed the motivation for us to develop the STDDP learning rule.

In this experiment, all the 512k components were configured to work in the STDDP adaptor mode. This experiment will fine tune all the axon delays from these 512k STDDP adaptors such that they will be evenly distributed from 0 ms to 15 ms. The axonal delays were initialised to be 0 ms and 15 ms (in the ratio of 50% to 50%). We used a paired-pulse protocol: a single pair of pre- and post-synaptic spikes is sent to each of the adaptors periodically (every 16 ms). During each period, each adaptor will receive one and only one pre-synaptic spike, the arrival time of which is set to be time step 1. Additionally, during each period, each adaptor will receive one and only one post-synaptic spike, the arrival time is between time steps 1 and 16. These spike pairs remain the same in each period. In each period, for each adaptor, a delay adaptation is performed if the axonal delay has not been tuned to the desired delay.

We have conducted the experiment with both modification rules. For the linear proportional modification rule, we use *A*^+^ = *A*^−^ = 1 throughout. Hence, theoretically, after one period (16 ms), all axonal delays will be fine-tuned to the desired values, which are evenly distributed from 0 to 15 ms. For the fixed step modification rule, we use *step* = 1 throughout. Hence, it will take 16 periods (256 ms) for all the axonal delays to be fine-tuned.

**FIGURE 13:**
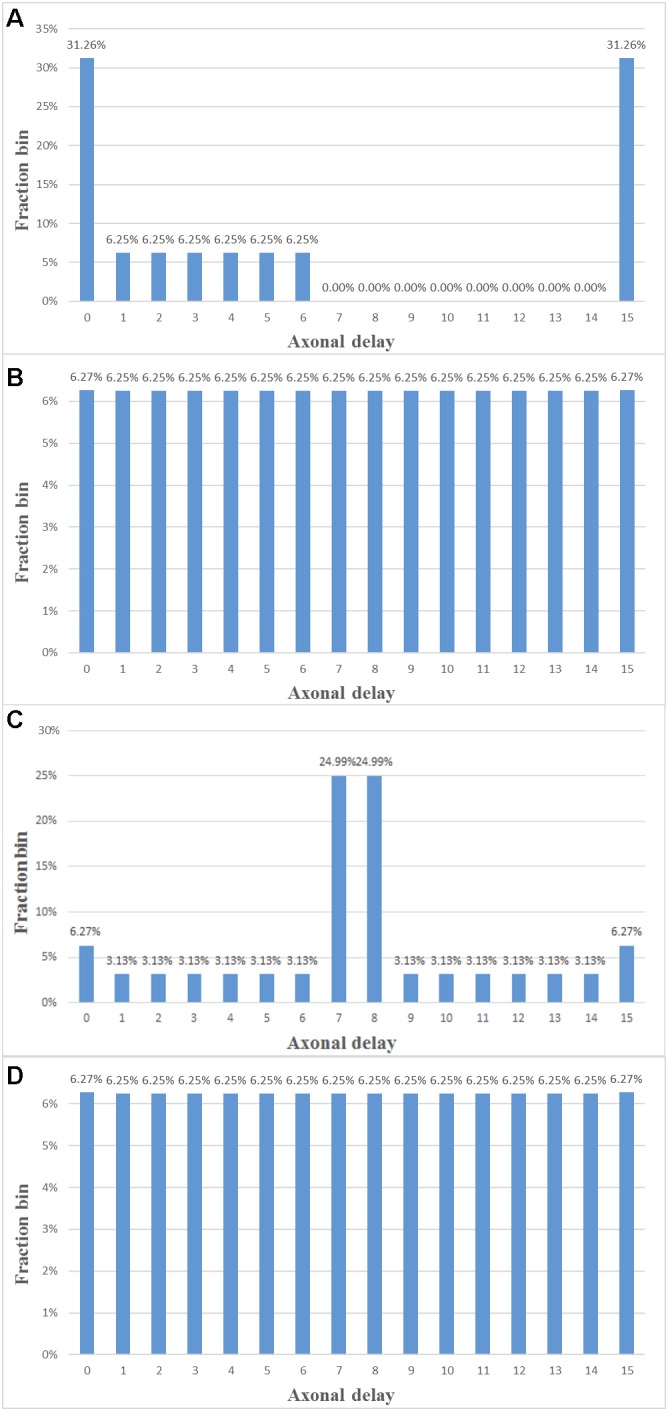
Polychronisation experiment. **(A,B)** Delay distribution after 8 and 16 ms of STDDP with the linear proportional modification rule. **(C,D)** Delay distribution after 128 and 256 ms of STDDP with the fixed modification rule (the adaptation step is set to 1 here).

Measurements on the FPGA confirmed the theoretical analysis. **Figure 13** shows the delay distribution in the middle of the experiment and the final delay distribution for both modification rules. In **Figure 13C**, after 128 updates of the STDDP, most axonal delays have been tuned to 7 ms or 8 ms, since the axonal delays were initialised to be 0 and 15 ms (in the ratio of 50% to 50%). As all the axonal delays have been fine-tuned to the desired values, we can conclude that the system has performed the polychronisation experiment successfully.

### Hardware Evaluation

The measurements from the FPGA prototype system demonstrate the functionality of the neural engine. To provide a quantitative hardware evaluation, particularly of the area efficiency and power efficiency, we implemented one neural engine (not the whole prototype system) using TSMC 28 nm (HPC) technology using the Cadence digital design flow. The power consumption was estimated using switching activity with Cadence Innovus. From this manufacturing process, we used the ARM physical library and with the smallest available standard cells with ultra-low threshold voltages (ULVT). For the SRAMs, we used the ARM high-density single-port SRAMs, the largest one of which has a size of 1 M bit (2^20^ bits, 8k × 128 bit). By putting two of them together, we implemented the dual-port SRAM in the neural engine, which has 16 physical components and uses a time-multiplexing rate of 2k × 8 = 16k, thus consists of 16 × 16k = 256k (2^18^) TM components using 2 M bits SRAMs. These choices were to achieve high area and power efficiency.

**FIGURE 14:**
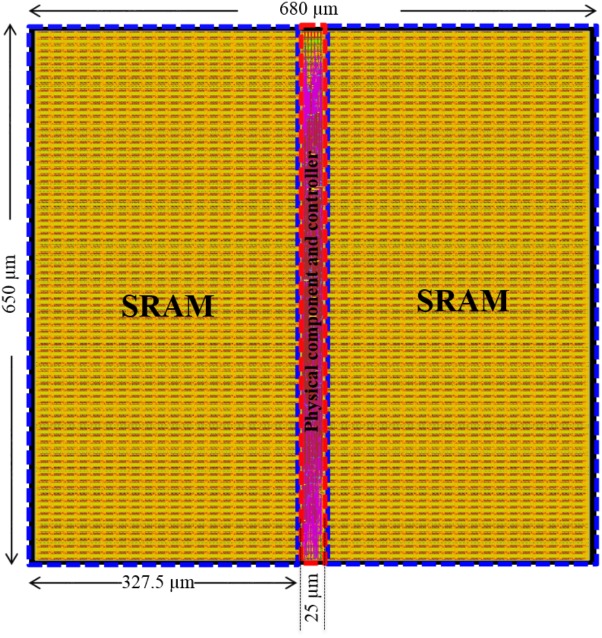
Neural engine place and route. To achieve the maximum utilization of the hardware resources, the engine was implemented with two high density single-port SRAMs (1 M bit each, in the blue rectangles). The physical component and the controller were placed between the SRAMs such that the routing can be easily performed. The utilization of the silicon area is 98.7 and 70.5% with and without SRAMs, respectively.

**Figure 14** shows the results of the place and route. The post place-and-route core size is 650 μm × 680 μm = 0.44 mm^2^ (core utilization = 98.7%), with an estimated power consumption of 37 mW at 0.9 V supply voltage and a clock frequency of 2.5 GHz. Each component occupies 1.68 μm^2^, of which 1.62 μm^2^ is the 8-bit SRAMs. Only 0.06 μm^2^ (∼3.5%) out of 1.68 μm^2^ is used for the reconfigurable modules, which are capable of performing three different functions. It is undoubtedly true that reconfigurable modules are flexible but usually involve more hardware design costs due to the design complexity. But by leveraging the time-multiplexing approach and state-of-the-art CMOS technologies, this overhead (hardware cost) has been reduced to a negligible level. In other words, implementing each individual function separately is not likely to significantly improve the area efficiency of this neural engine. If the time-multiplexing were not extensively used, the use of the reconfigurable modules need to be further investigated. This area efficiency demonstrates the necessary and advantages of the use the time-multiplexing approach.

The power efficiency of the neural engine is 37 mW/256k ≈ 141 nW per component. Since this engine is capable of running at 2.5 GHz and the time-multiplexing rate is 16k, each component will have a time slot of up to 1 ms/(400 ps × 16k) = 156 clock cycles. The engine is capable of performing up to 2.5 G × (155/156) × 16 ≈ 40 G SOPs per second, yielding a power efficiency of ∼0.92 pJ per SOP. The area and power efficiency demonstrate that our neuromorphic engine is a high-performance and scalable design.

## Discussion and Future Work

Our work fills in a vacant niche along the spectrum of neuromorphic platform solutions between integrated specialized hardware for diverse spiking neural network components and software emulation on general purpose hardware, e.g., CPUs and GPUs. This is a previously unexplored point of trade-off that inspires theoretic insights into algorithmic designs of spiking neural networks and warrants hardware investigations.

To the author,s acknowledgment, our neural engine is the first and only hardware neuromorphic implementation that is capable of working as a LIF neuron, a learning synapse, and an axon. In the SpiNNaker (Furber et al., 2014) project, ARM processors run software neural models and are programmable. In theory, it should be capable of trading off the number of the neurons and the synapses. However, it is a software implementation, which performs numerical simulations, and hence is not comparable to our hardware implementation that emulates the LIF neurons and plastic synapses on silicon directly.

To compare our work with state-of-the-art neuromorphic implementations, a performance and specification summary of them is provided in **Table 6** [adapted and completed from Frenkel et al. (2018)]. On the left are the mixed-signal designs (Schemmel et al., 2010; Benjamin et al., 2014; Park et al., 2014; Qiao et al., 2015; Mayr et al., 2016; Moradi et al., 2018), digital designs (Seo et al., 2011; Merolla et al., 2014; Davies et al., 2018; Frenkel et al., 2018) together with this work on are on the right. Toward large-scale spiking neuromorphic platforms, the key figures of merit are density, flexibility, synaptic plasticity, and energy consumed per SOP.

**Table 6:** Comparison of with the state of the art of spiking neuromorphic circuits, adapted and completed from (Frenkel et al., 2018).

	HICANN	NeuroGrid	ROLLS	DYNAPs	IFAT	Mayr et al., 2016	Seo et al., 2011	TrueNorth	Loihi	Odin	This work
Implementation	Mixed-signal	Mixed-signal	Mixed-signal	Mixed-signal	Mixed-signal	Mixed-signal	Digital	Digital	Digital	Digital	Digital
Technology	180 nm	180 nm	180 nm	28 nm	90 nm	28 nm	45 nm	28 nm	14 nm	28 nm	28 nm
Area of a neurosynaptic core [mm^2^]	26.3	168	51.4	7.5	0.31	0.36	0.8/1.15	0.095	0.4	0.086	0.44
Neurons per core	512	64k	256	256	2k	64	256	256	max. 1024	256	256k^†^
Synaptic weight storage	4-bit SRAM	Off-chip	Capacitor	12-bit CAM	Off-chip	4-bit SRAM	1-bit/4-bit SRAM	1-bit SRAM	1- to 9-bit SRAM	(3+l)-bit SRAM	3-bit SRAM
Embedded online learning	STDP	No	SDSP	No	No	SDSP	Probabilistic STDP	No	Programmable	SDSP	STDP STDDP
Synapse per core	112k	–	128k	16k	–	8k	64k	64k	1M to 114k (1-to 9-bit)	64k	256k^†^
Neuron model	Adaptive exponential IF	Adaptive quadratic IF	Adaptive LIF	Adaptive LIF	2-compartment LIF	Adaptive exponential IF	Configurable LIF	Configurable LIF	Adaptive LIF	Phenomenological	2-compartment LIF
Time constant	Accelerated	Biological	Biological	Biological	Biological	Bio to accel	Biological	Biological	N/A	Bio to accel	Bio to accel
Neuron core density [neur/mm^2^]^∗^	19.5	390	5	34	6.5k	178	827/575	2.6k	Max. 640	3.0k	582k^†^
Synapse core density [syn/mm^2^]^∗^	4.3k	–	2.5k	2.1k	–	22.2k	207k/144k	673k	640k to 71k	741k	582k^†^
Supply voltage	1.8 V	3.0 V	1.8 V	1.3 V-1.8 V	1.2 V	0.75 V, 1.0 V	0.53 V–1.0 V	0.7 V–1.05 V	0.5 V–1.25 V	0.55 V–1.0 V	0.9 V
Energy per SOP	N/A	941 pJ	77 fJ	134 fJ–417 fJ	22 pJ	>850 pJ°	N/A	26p at 0.775 V	>23.6 pJ at 0.75^‡^	9.8 pJ at 0.55 V	0.92 pJ

*^†^Assuming all the components have been configured for neurons or synapses. ^∗^Neuron/synapse core density of digital designs is normalized to a 28 nm technology node. Normalization is not applied to mixed-signal designs as the analog parts require redesign to compensate for performance degradation during technology scaling. °Represents a lower bound, estimated by considering the synaptic weights at half their dy ^‡^Represents a lower bound as it includes only the contribution of the synaptic operation, without taking into account the cost of neuron update and learning engine update.*

Our future work will focus on scaling up the network that we have presented here. As a fully digital implementation, the neural engine is a scalable design. The number of physical components, i.e., the ones that can be activated simultaneously, will increase linearly with the number of available logic gates, which are usually the bottleneck for high performance FPGA designs. But in our system, the design of the physical component costs only a few logic gates and plenty of resources are left for additional physical components or other systems modules.

High-end FPGAs, such as Xilinx’s Virtex UltraScale+ (XCVU09P of a Xilinx VCU118 kit) have ∼400 M bits on-chip SRAMs. For a system running at 400 MHz, we can achieve 4k TM components with one physical engine (the time slot keeps the same, 100 clock cycles) for a sub-millisecond time resolution. Each engine has 16 physical components and thus 16 × 4k = 64k TM components using 512k bits SRAMs. We can implement 400 M/512k = 800 neural engines, yielding 50 M TM components. Based on the above calculations and analysis, we can conclude that it is practical to scale the proposed system up to a system with 50 M TM components on a commercial off-the-shelf high-end FPGA.

Another future improvement will be to accommodate more complicated learning rules. Since this paper is only for proof of concept, we made an arbitrary design choice: for both the STDP and STDDP learning rule, the time window (ramp) needs to become “inactive” before it can be started again. This means the first spike dictates when the learning window begins and ends. The same spike train might have disparate learning behaviors with a potentially small modification in the length of the learning window. This could affect the robustness and efficiency of the learning system, since a small perturbation in a minor parameter might result in different outcomes. Multiple methodologies have been developed in addressing such learning “conflicts,” one of which is a resource-based STDP (Hunzinger et al., 2012). These learning rules are hardware friendly, as they introduced few additional state variables. Due to its unique architecture, the proposed neural engine can be easily adjusted to such learning rules.

## Author Contributions

RW and AvS conceived the idea and wrote the paper. RW developed the hardware implementation, and designed and conducted the experiments.

## Conflict of Interest Statement

The authors declare that the research was conducted in the absence of any commercial or financial relationships that could be construed as a potential conflict of interest.
